# Protein folding on the ribosome studied using NMR spectroscopy

**DOI:** 10.1016/j.pnmrs.2013.07.003

**Published:** 2013-10

**Authors:** Christopher A. Waudby, Hélène Launay, Lisa D. Cabrita, John Christodoulou

**Affiliations:** Institute of Structural and Molecular Biology, UCL and Birkbeck College, London WC1E 6BT, United Kingdom

**Keywords:** Nuclear magnetic resonance, Protein folding, Co-translational, Ribosome, Sensitivity

## Abstract

NMR spectroscopy is a powerful tool for the investigation of protein folding and misfolding, providing a characterization of molecular structure, dynamics and exchange processes, across a very wide range of timescales and with near atomic resolution. In recent years NMR methods have also been developed to study protein folding as it might occur within the cell, in a *de novo* manner, by observing the folding of nascent polypeptides in the process of emerging from the ribosome during synthesis. Despite the 2.3 MDa molecular weight of the bacterial 70S ribosome, many nascent polypeptides, and some ribosomal proteins, have sufficient local flexibility that sharp resonances may be observed in solution-state NMR spectra. In providing information on dynamic regions of the structure, NMR spectroscopy is therefore highly complementary to alternative methods such as X-ray crystallography and cryo-electron microscopy, which have successfully characterized the rigid core of the ribosome particle. However, the low working concentrations and limited sample stability associated with ribosome–nascent chain complexes means that such studies still present significant technical challenges to the NMR spectroscopist. This review will discuss the progress that has been made in this area, surveying all NMR studies that have been published to date, and with a particular focus on strategies for improving experimental sensitivity.

## Highlights

• Despite size of ribosome, flexible regions can be observed by solution-state NMR.
• Development of NMR methods for the direct observation of co-translational folding.
• We review all NMR studies of ribosome–nascent chain complexes published to date.
• Large, dilute and unstable nature of samples presents a challenging target for NMR.
• In-depth review of strategies to improve experimental sensitivity.

## Graphical abstract

**Figure:**
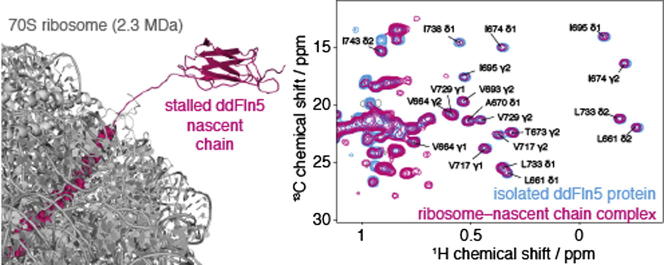

## 1 Introduction

The process by which a nascent polypeptide acquires its native folded structure after emerging from the ribosomal exit tunnel has been the subject of intense research since the pioneering observations of Anfinsen and Levinthal [1–6]. The ‘protein folding problem’ is not one of purely scientific interest however, for the failure of proteins to reach a stable structure may result in the population of misfolded or aggregated species that are implicated in a very wide range of pathological disorders [7]. In some cases this may be the result of mutations that destabilize the native state, while other mutations may stabilize intermediates or transition states in the pathway to aggregated species such as amyloid fibrils. However, in many cases misfolding may occur in the absence of any apparent genetic or environmental factors, purely as a result of chance excursions into misfolded regions of the folding landscape. Such occurrences may give rise to late-onset disorders, such as Alzheimer’s and Parkinson’s diseases. Clearly, there is an evolutionary incentive to minimize such events, but only to the extent that they impinge upon the process of natural selection. With the large increase in human lifespan in the past several centuries, no selective pressures can yet have acted, and in this regard such diseases have been described as ‘post-evolutionary’ [8]. In all cases, however, by developing our understanding of folding landscapes, energetically and structurally, we may hope to rationally develop strategies to destabilize misfolded states and guide folding to the desired native state.

Protein folding is most commonly studied using isolated proteins in dilute aqueous solutions, either under equilibrium conditions (e.g. measurements of thermodynamic stability by reversible denaturation) or by studying relaxation kinetics following perturbations such as chemical denaturants, temperature or pressure. NMR spectroscopy may be used to follow folding in real-time, for example using stopped-flow methods to allow the characterization of intermediate states [9–11], but it has also proved to be a particularly powerful probe of protein folding at equilibrium, in the characterization of transient unfolding events through amide hydrogen exchange kinetics [12,13], or in the characterization of high-energy intermediate states through the analysis of relaxation dispersion phenomena [14]. Such measurements can characterize not only the thermodynamics of the excited state and the associated transition state, but also can determine structural parameters such as chemical shifts and residual dipolar couplings (RDCs) in the excited state. In combination with restrained molecular dynamics (MD) simulations, these may be used to develop detailed structural models of otherwise invisible intermediate states [15–18].

Within living cells, however, protein folding is not an equilibrium process, but may occur in a vectorial manner as the nascent polypeptide emerges from the ribosome, during translation, into the complex cellular environment [5,6,19]. The co-translational acquisition of folded structure has been demonstrated for a number of systems by a very wide range of biophysical, spectroscopic and imaging techniques, including functional assays [20–22], limited proteolysis [23], conformation-specific antibody recognition [24,25], assays of disulfide bond formation [26,27], FRET [28,29], time-resolved fluorescence anisotropy [30], cryoelectron microscopy [31,32], atomic force microscopy [33], single molecule force spectroscopy [34,35] and, of particular interest to the present review, NMR spectroscopy [36–40]. The tethering of the nascent chain to the ribosome during synthesis may affect the folding process in several ways. The simple presence of a nearby surface may result in entropic, excluded volume effects on the thermodynamics of the folding process, while interactions with the surface may preferentially stabilize folded or unfolded states, particularly given the high local concentrations resulting from tethering [34]. Such effects may perturb the kinetics or thermodynamics of the folding reaction, potentially altering the folding pathway from that that would be observed in equilibrium studies of the isolated protein. However, the vectorial nature of protein synthesis will also give rise to an energy landscape that evolves as a function of chain length [41]. This may create ‘shortcuts’ through the energy landscape, allowing folding to proceed in a more efficient, directed manner and reducing the likelihood of populating misfolded states. This is a fundamentally non-equilibrium process, and one that is only accessible to exploration in co-translational systems [6,42].

The mechanism of co-translational folding is also of great interest in protein systems subject to topological constraints. A survey in 2006 determined that 0.8% of proteins in the Protein Data Bank contained a knot in their fold [43]. While undoubtedly rare, the existence of such knotted proteins presents interesting and extreme cases that challenge theoretical models of protein folding [44–47]. A prominent question is to understand the process of knot formation: can this occur co-translationally? Certainly, the presence of the ribosome places severe constraints on knot formation via the C-terminus. Again however, this information is not accessible through reversible folding studies, for in at least some cases knots have been shown to persist in highly denatured states [48]. Non-equilibrium kinetic studies of the first folding events following synthesis and release have begun to address this question [49], but beyond a small number of particular cases, much remains to be understood about the conditions under which knot formation may occur co-translationally.

**Fig. 1:**
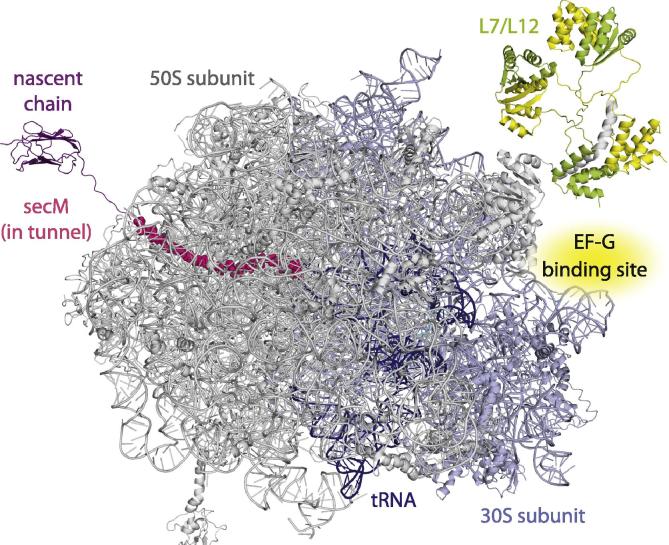
Crystal structure of the *E. coli* 70S ribosome [53], showing complexed tRNA molecules aligned from a crystal structure of *Thermus thermophilus*[56], L7/L12 proteins modeled using NMR structures of the isolated proteins docked onto the L10 subunit [57–59], and a nascent polypeptide modeled upon a cryo-EM structure of a TnaC nascent chain [31].

What contributions, then, might NMR spectroscopy be able to make to the questions outlined above? The power of NMR lies in the ability to provide rich information on both protein structure and dynamics and, in contrast to most alternative methods, the ability to simultaneously resolve many spin systems using multidimensional spectroscopy supplies a very large number of probes, resulting in a description of the entire system with near atomic resolution. It is clear that solution-state NMR spectroscopy will not compete with X-ray crystallography and cryo-electron microscopy in the structural characterization of the rigid 2.3 MDa core of the 70S ribosome (Fig. 1) [50–53], or in studies of structural preferences within the ribosomal exit tunnel [31,32]. On the surface of the ribosome however, and outside the tunnel, the flexibility of polypeptide chains gives rise to structural heterogeneity that has so far defied structural characterization by either X-ray crystallography or cryo-electron microscopy. It is here that NMR spectroscopy has proved to be a highly complementary technique, for the flexibility of such regions results in a reduction in their effective rotational correlation time, giving rise to sharp resonances despite the large size of the core ribosome particle. This observation led in 2004 to the high-resolution characterization of the structure and dynamics of the L7/L12 ribosome stalk region [54,55], which due to its flexibility was not observable in crystallographic studies of the ribosome. These initial findings stimulated the investigation of the structural and dynamical properties of ribosome-bound nascent chains during co-translational folding by NMR and, in 2007, we reported the first observation of a ribosome–nascent chain complex (RNC) by solution-state NMR spectroscopy [36].

However, while the potential of NMR to provide rich structural and dynamic information on co-translational protein folding is clear, it is still a subject that poses a large number of technical challenges. The preparation of RNCs, either through *in vitro* or *in vivo* methods, is a technically challenging process that requires the production of samples in which the ribosomes are programmed to uniformly arrest during translation a nascent chain of a given sequence. The production of RNCs for NMR studies must be carefully optimized in order to selectively isotopically label the nascent chain only, while delivering very high yields of stable and homogeneous complexes that are of a maximum nascent chain occupancy (the fraction of ribosomes carrying an attached nascent chain). Moreover, once produced, RNC samples are proving to be only moderately stable, with typical lifetimes of hours–days before the nascent chain is released from the complex or the ribosome particle itself starts to dissociate. Continual assessment of the sample integrity is therefore required throughout the data acquisition period to ensure that the measured signal is derived exclusively from ribosome-bound nascent chains. Thus, given the limited sample lifetimes, in combination with the maximum working concentrations possible for ribosomes (ca. 10 μM) and their large molecular weight (2.3 MDa), low sensitivity is the dominant characteristic of RNC NMR and experiments must be carefully designed to maximize the data obtained from each sample.

In this article, we will initially review the use of solution-state NMR spectroscopy for the study of the structure and dynamics of isolated ribosomes (Section 2). These observations were instrumental in demonstrating the feasibility of high-resolution studies of flexible domains within very large molecules, and led directly to studies of ribosome–nascent chain complexes by NMR. As outlined above however, there are many biochemical and spectroscopic challenges associated with the preparation and study of these large, unstable and low concentration samples by NMR, and these are discussed in detail in Section 3. We then review all NMR studies of RNCs that have been published to date (Section 4), considering both the biochemical and spectroscopic approaches taken in each case, and also the biological information that has been gleaned from these studies. Finally, we close by discussing the potential for the field in the future (Section 5).

## 2 Solution-state NMR studies of the ribosome

The first studies of ribosome structure by solution-state NMR spectroscopy were reported in 1980 and revealed that, despite the 2.3 MDa molecular weight of the ribosome (with a rotational correlation time of 2.5 μs at 298 K [60]), sharp resonances were apparent in the 1D ^1^H spectrum of the 70S particle [61]. This indicates the existence of domains having significant mobility and effective correlation times one or two orders of magnitude reduced from that of the whole complex. Both 30S and 50S subunits were determined to contribute to the observed signal, although no resonances could be detected from purified rRNA components [61]. Similar resonances were later also observed from the large 60S subunit of the eukaryotic ribosome [62]. By selective exchange and removal of subunits, observed signals were traced to the S1 protein in the 30S subunit and the L7/L12 proteins in the 50S subunit [62,63], although the intensities of S1 resonances decrease significantly in the 70S particle. Moreover, addition of the elongation factor EF-G, which binds primarily to the 23S rRNA (Fig. 1), broadened the resonances of L7/L12 beyond detection, indicating that the dynamics of the stalk complex are quenched in the presence of the elongation factor [64]. NMR studies of the isolated L7/12 protein subsequently identified a conserved region on the surface of the C-terminal domain that interacts with EF-G, and also initiation factor 2 (IF2) and elongation factor Tu (EF-Tu), with millimolar affinity which, given the high local concentration of these translation factors when bound to the ribosome, results in large populations of the bound state on the ribosome [65].

**Fig. 2:**
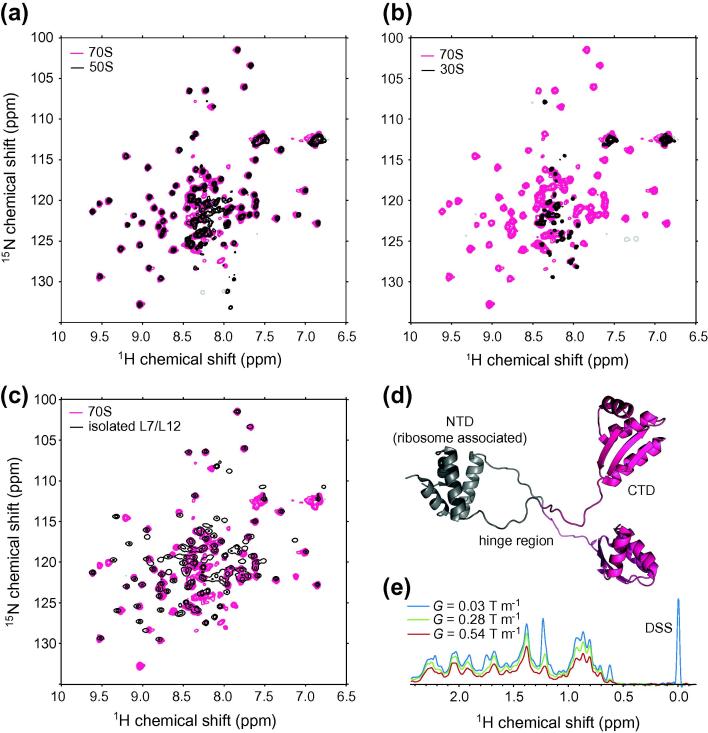
^1^H,^15^N HSQC spectrum (700 MHz, 298 K) of 70S ribosomes overlaid with spectra of the (a) 50S and (b) 30S subunits, and (c) with the isolated L7/L12 protein from the 50S subunit. (d) NMR structure of the isolated L7/L12 dimer [57], colored magenta to indicate residues observed in the ribosome-associated state. (e) ^1^H STE PFG diffusion measurements of 70S ribosomes (700 MHz, 298 K, 100 ms diffusion delay, 4 ms bipolar gradients with strengths as indicated, data acquired at UCL Biomolecular NMR Facility) for determination of the hydrodynamic radius of observed species and assessment of ribosomal integrity.

These early results laid the path for later studies of the ribosome itself using high-resolution heteronuclear NMR methods. Well-resolved ^1^H–^15^N HSQC spectra can be obtained from isotopically labeled 70S ribosomes, and the observed resonances overlay closely with those of the individual 30S and 50S subunits (Fig. 2a and b) and, in particular, with the L7/L12 protein of the 50S subunit (Fig. 2c) [54,55]. Resonances are observed from the hinge and C-terminal domains (residues 42–120) of L7/L12 (Fig. 2d), and while there are small chemical shift perturbations in the hinge region, no significant chemical shift changes are observed in the C-terminal domain relative to the free protein, indicating that its structure is unchanged in the ribosome-associated form [55]. A key methodological development was the application of diffusion NMR measurements to verify the hydrodynamic radius of the observed species, providing direct confirmation that the spectra originate from ribosome-associated species rather than dissociated or degraded ribosomes (Fig. 2e) [54]. As discussed further below (Section 3.4), this is also an essential control measurement in the observation of RNC samples [36].

Analysis of the intensity of the L7/L12 resonances shows that two of the four copies present have sufficient mobility to be observed by solution-state NMR [54,55]. However, a small increase in the ^15^N linewidth from an average of 6.3 ± 0.8 Hz to 9.3 ± 1.6 Hz was observed in the ribosome-bound form, indicating that rotational diffusion is still partially restricted [55]. ^15^N nuclear spin relaxation measurements of the ribosome-bound form showed *T*_2_ relaxation times of 30–40 ms in the structured core (residues 52–120), while those in the hinge region (residues 42–52) had somewhat slowed relaxation, with *T*_2_ values of 70–90 ms [54]. An averaged apparent rotational correlation time of 13.6 ns was calculated, larger than that expected for the protein in the free state but certainly much less than that the value of 2.5 μs measured for the entire 70S particle [60], again indicating that tumbling of L7/L12 is largely decoupled from that of the ribosome. Finally, analysis of the relaxation data using a model of anisotropic rotational diffusion provided orientational restraints allowing a molecular model of this flexible region to be docked into a crystal structure of the core ribosome particle. Importantly, the resulting dynamic model is able to rationalize the interaction of L7/L12 with several other components of the ribosome including L10, L11 and EF-G, as observed in a series of cryoelectron and immunoelectron microscopic studies [66–69], providing an excellent example of the complementarity between NMR spectroscopy and other structural techniques. Moreover, these high-resolution studies of L7/L12 were instrumental in demonstrating the feasibility of NMR studies of co-translational folding in ribosome–nascent chain complexes.

## 3 Technical aspects of the NMR of ribosome–nascent chain complexes

### 3.1 Choice of isotopic labeling scheme

Central to the study of RNCs by NMR is the ability to generate selectively isotopically-labeled and homogeneously stalled, ribosome-bound nascent chains. The selection of an isotopic labeling scheme for NMR studies of RNCs depends primarily on the folded or unfolded nature of the nascent chain species being targeted. The majority of RNC studies reported to date have used uniform ^15^N (or ^15^N/^13^C) labeling, allowing the observation and characteriastion of both folded and disordered states in a single spectrum [36,38–40]. However, significantly greater sensitivity may be obtained using a ^13^C labeling scheme to observe methyl groups, due to the greater proton concentration (three times that of amide groups) and to the favorable relaxation properties resulting from rapid rotation about the threefold symmetry axis [37].

In the future, RNC studies may be expected to benefit from the application of more advanced isotopic labeling schemes, which combine perdeuteration of samples with the selective introduction of ^13^CH_3_ labels on sidechain methyl groups, most commonly on I, L and V residues, resulting in substantial reductions in transverse relaxation rates. Such labeling schemes, in combination with ‘methyl-TROSY’ ^1^H–^13^C HMQC experiments that avoid the mixing of slow and fast relaxing coherences [70], have enabled the high-resolution characterization of extremely large species such as the 20S proteasome (MW 670 kDa, *τ*_c_ approximately 180 ns at 65 °C) [71,72], and while these methods have not yet been applied to the study of RNCs, the potential gains in sensitivity and resolution appear to be extremely promising. However, a major limitation of ^13^C-based experiments is the limited chemical shift dispersion. This is particularly problematic in the case of partially disordered states that are likely to be present in emergant nascent chains, and so when such species are being investigated it is preferable to take advantage of the large dispersion of the amide nitrogen chemical shift using a simple ^15^N labeling scheme.

### 3.2 Preparation of ribosome–nascent chain complexes

The preparation of homogeneous and stable RNC samples in a reliable, cost-effective manner, and on the nmol scale required for NMR studies, is perhaps the most significant technical challenge associated with this field. Additionally, the production of homogeneous samples of programmed ribosomes requires that translation must be arrested specifically at a given point in the protein sequence. Two main approaches have emerged to fulfill this need, each with different strengths: *in vitro* transcription–translation systems, in which cell extracts or reconstituted ribosomes are used to synthesize the nascent chain in a controlled, cell-free environment; and *in vivo* approaches, in which stalled RNCs are generated within living cells directly, before being harvested and purified.

The initial RNC samples studied by NMR were produced using an *Escherichia coli in vitro* coupled transcription–translation system, in combination with a linearized DNA template that lacks a stop codon to terminate translation [36,37]. In the absence of a stop codon, release factors cannot bind and the C-terminus of the nascent chain remains covalently attached to the P-site tRNA by the aminoacyl ester bond. With unlabeled ribosomes in the reaction mixture, the use of isotopically labeled amino acids allows for the translation of selectively labeled nascent chains, and together with a non-stop sequence, enables the nascent chains to remain bound to the ribosome. This strategy has the particular advantage that NMR-silent ribosomes are obtained, and therefore the resulting spectra are free from overlap with resonances from the L7/L12 stalk complex. The recently developed PURE (protein synthesis using recombinant elements) cell-free approach [73,74] also allows, in principle, control over all aspects of translation, including the presence or absence of auxiliary factors such as the ribosome-associated chaperone trigger factor (TF), release factors and other quality control mechanisms (e.g. tmRNA tagging in trans–translation) that release stalled ribosomes. However, *in vitro* approaches are typically extremely costly in terms of routine usage at the scales required for NMR spectroscopy, and together with the prospect of generating RNCs within their natural environment, this has stimulated the development of alternative *in vivo* approaches to sample production.

An *in vivo* approach for generating RNCs presents unique challenges compared to *in vitro* strategies, as there is less control of the ability to manipulate both translational arrest and the selective labeling of the nascent chain. To enable homogeneous translational arrest *in vivo*, protein motifs are introduced at the C-terminus of a DNA sequence that interact directly with the ribosome exit tunnel. Such sequences have been derived from proteins that modulate the translation of a downstream gene, providing an additional point of regulation for the control of gene expression [75]. For example, transient translational arrest of the secretion monitor protein in *E. coli* (SecM) regulates the translation of the downstream gene SecA, a protein involved in peptide secretion, according to the activity of the secretory system. Active translocation of SecM via an upstream signal peptide relieves translational arrest, whereupon the protein is exported to the periplasm and degraded. However, if secretion is impaired, stalling of the nascent SecM polypeptide induces a change in the secondary structure of the downstream mRNA to reveal a hidden initiation site for the SecA gene, and in this way the levels of SecA, and the activity of the secretory system, can be continually tuned to meet fluctuating demand [75,76]. The introduction of the 17 amino acid motif (F^150^XXXXWIXXXXGIRAGP^166^) derived from SecM to selectively stall nascent chains has proved to be instrumental in the development of RNC studies by NMR. Translational arrest is achieved from the presence of Pro166 within the P-site of the PTC, together with interactions made by a number of highly conserved residues within the SecM sequence with the ribosomal tunnel [76,77]. Selective stalling of nascent chains on *E. coli* ribosomes for structural studies using cryoEM has also been achieved using sequences derived from *E. coli* tryptophanase operon, *TnaC* [31], while sequences derived from the human cytomegalovirus and fungal arginine attenuator peptide have been used to stall eukaryotic ribosomes [78]. It is likely that these arrest sequences as well as others that have been reported recently [79] may also prove to be useful for future NMR studies of RNCs.

To achieve selective isotopic labeling of the RNCs *in vivo*, the culturing conditions of the *E. coli* are manipulated, such that during growth a medium is used which permits the production of unlabeled ribosomes. The cells are then transferred into an isotopically rich medium in which translation takes place. Varying combinations of growth/expression media have been reported [39,40], though we find that high yields and reproducibility of RNC production required for NMR studies are achieved with the use of media compositions and growth conditions that can sustain high-cell density growth [38]. Following expression, the *in vivo* derived RNCs are subject to rigorous purification to recover homogeneous and highly programmed ribosomes, to ensure the strongest possible nascent chain NMR signal. Typical purification strategies involve a three-step strategy including sucrose cushions to isolate crude ribosomal material, affinity purification in which a tag (e.g. 6xHis or Strep) is used to isolate nascent chain containing ribosomes, and a sucrose density gradient to isolate intact monosomes. Finally, a combination of biochemical and NMR strategies are used to evaluate the integrity of the sample before and during acquisition. In particular, the ribosome concentration may be determined by UV absorption at 260 nm (*ε* = 4.17 × 10^7^ M^−1^ cm^−1^) [54], while the attachment of the nascent chain may be verified by Western blotting directed against N-terminal purification tags [38,40].

### 3.3 NMR spectroscopy at very low concentrations

A defining feature of RNC NMR is the low sample concentration. In contrast to solution state NMR studies of isolated proteins or nucleic acids, which are typically carried out at concentrations of at least 100 μM and often several mM, ribosomes are limited to a concentration of 10 μM (we note however that given the large molecular weight of the ribosome, this still corresponds to a concentration of 23 mg/mL). The nascent chain concentration itself is also critical, and given the unstable and often complex behavior of RNC samples, the fraction of ribosomes harboring a nascent chain can change during the course of NMR data acquisition as the sample degrades. Clearly therefore, it is essential to optimize the acquisition process in order to maximize the signal to noise ratio (SNR) of the acquired data. Indeed, an entirely different mindset is required when compared to typical biomolecular NMR experiments, for given the limited lifetime of RNC samples it is simply not possible to compensate for inadequate signal by acquiring additional scans: it is essential that experiments are highly optimized before the experiments are begun. Therefore, the following four sections are dedicated to a review of methods for maximizing experimental sensitivity, through the spectrometer hardware and the design of pulse programs.

#### 3.3.1 Hardware developments

At the level of the spectrometer hardware, the SNR in an experiment is described by:

(1) $$\text{SNR}\propto \frac{n\gamma_{\text{e}}\sqrt{B_{0}^{3}\gamma_{\text{d}}^{3}t}}{\sqrt{R_{\text{S}}(T_{\text{S}}+T_{\text{A}})+R_{\text{C}}(T_{\text{C}}+T_{\text{A}})}}$$

where *n* is the quantity of spins in the detector, *t* is the total experimental time, *B*_0_ is the magnetic field strength, *γ*_d_ (*γ*_e_) is the gyromagnetic ratio of the nucleus being detected (excited), *R*_S_ and *R*_C_ are the resistances of the sample and the detector coil, and *T*_S_, *T*_C_ and *T*_A_ are the temperatures of the sample, coil and preamplifier [80]. Given the low concentration and limited stability of RNC samples, there is little opportunity to increase SNR through increasing *n* and *t*, and ^1^H-based experiments already provide the greatest possible sensitivity with regard to the *γ*^5/2^ scaling. However, hardware developments have had, and will continue to have, a very significant impact on the field. Given the scaling of SNR with $B_{0}^{3/2}$ for non-lossy samples, increases in spectrometer field strength provide large gains in sensitivity and, for example, the strongest field strength commercially available at the time of writing, 23.5 T (1000 MHz), would provide a 71% increase in sensitivity compared to the 16.4 T (700 MHz) spectrometers that have been used for several RNC studies to date [38,40]. However, for lossy samples the gain with field strength may be less.

The advent of cryogenic probe technology has revolutionized the capacity to study low concentrations of biomolecules: by reducing resistance and thermal noise in the coil and preamplifier (*R*_C_, *T*_C_ and *T*_A_ in Eq. (1)), the SNR may be increased three or fourfold over a conventional probe [80]. At this point, sensitivity is limited primarily by rf power dissipation within the sample (*R*_S_*T*_S_ term in Eq. (1)), which has contributions from both ionic and dielectric conductivity. Where possible, this should be minimized by optimization of the buffer composition, such as reducing the ionic strength or using low-conductivity salts [81,82], but in the case of RNC samples the buffer components required to maintain the integrity of the ribosome particle places conflicting demands on the buffer composition. Dielectric power dissipation is not however uniform across the sample volume, but is dominated by electric-field ‘hotspots’ at the edges of the sample parallel to the *B*_1_ field [83]. An alternative possibility is therefore to use (without concentration of the sample) NMR tubes with smaller diameters [84], or shaped tubes designed and oriented to minimize electric-field hotspots [1–6,83,85]. Despite the smaller total quantity of spins in such tubes, at moderate ionic strengths genuine improvements in SNR may be obtained.

#### 3.3.2 Optimal sampling for two-dimensional NMR experiments

Two-dimensional (2D) NMR spectroscopy is the most basic component of biomolecular NMR capable of providing residue-specific probes of structure and dynamics. The indirect dimension of 2D experiments is encoded as a series of *N* points, with the chemical shift evolution period incremented by a dwell time, Δ*t*, between each point, resulting in a total acquisition time *T*_aq_ = *N* · Δ*t*. The dwell time has a reciprocal relation to the spectral width, while the frequency resolution is related to the total acqusition time, Δ*f* = 1/*T*_aq_. Both the spectral width and the number of increments must be set prior to acquisition, and this choice has important consequences for the sensitivity of the experiment.

**Fig. 3:**
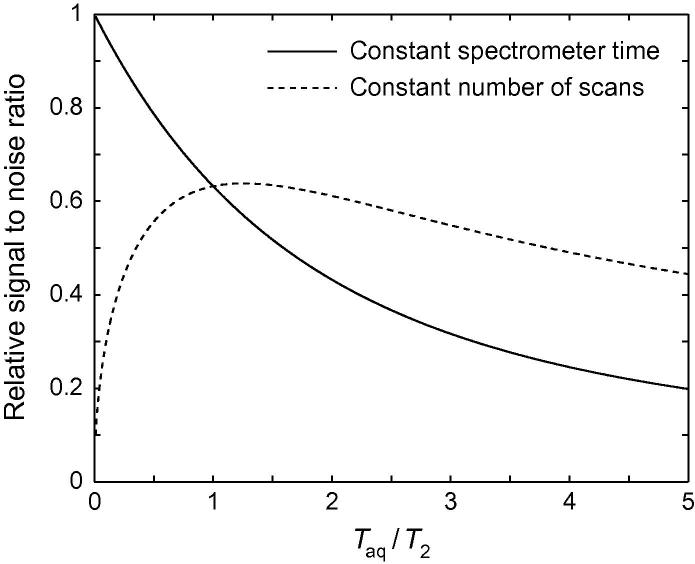
Relative sensitivity of 2D NMR experiments as a function of the acquisition time, *T*_aq_, relative to the tranverse relaxation time, *T*_2_ (Eqs. (2) and (3)).

The signal intensity, *S*(*t*), is related to the indirect evolution time, *t*, and the transverse relaxation time, *T*_2_, of the spin system being observed: *S*(*t*) ∝ exp(−*t*/*T*_2_). In the common case where the sample is stable and a constant number of scans are acquired at each point, the total spectrometer time is directly proportional to the acquisition time, and may be increased without restriction. The measurement noise is therefore proportional to $\sqrt{T_{\text{eq}}}$, and so the net signal-to-noise ratio is described by Eq. (2) [7,86]. Plotted in Fig. 3 (dashed line), it may be observed that the SNR improves as the acquisition time is increased, up to a broad maximum at *T*_aq_ ≈ 1.26*T*_2_, beyond which point SNR gradually decreases.

(2) $$\text{SNR}\propto \frac{T_{2}}{\sqrt{T_{\text{aq}}}}[1-\exp(-T_{\text{aq}}/T_{2})]$$

In contrast, if the sample is unstable then optimizing sensitivity becomes a question of most effectively partitioning a limited amount of spectrometer time between indirect time points. In this case, as the total spectrometer time is constant, the noise is also constant and so the experimental sensitivity (signal-to-noise ratio) is proportional to the acquired signal only. For a given acquisition time *T*_aq_, this is given by:

(3) $$\text{SNR}\propto \frac{T_{2}}{T_{\text{aq}}}[1-\exp(-T_{\text{aq}}/T_{2})]$$

Eq. (3) is plotted in Fig. 3 (solid line), from which it is easily seen that SNR decreases monotonically with the acquisition time, and therefore that the sensitivity is also inversely related to the frequency resolution, Δ*f*. Thus, for unstable samples where spectrometer time is limited, the acquisition of multidimensional NMR data presents a fundamental trade-off between sensitivity and resolution. Therefore, to maximize sensitivity, the minimum required spectral resolution for an RNC spectrum should be carefully considered prior to the acquisition of RNC data, and the acquisition time (i.e. number of increments) adjusted accordingly. As an aside, we also note from Eq. (3) that for a given acquisition time the SNR is independent of the spectral width and therefore there is no sensitivity advantage in folding spectra (minimizing both the spectral width and the number of increments by careful aliasing of resonances). This result, applicable to sensitivity-limited acquisition, is in contrast to the more common situation of sampling-limited acquisition, where a reduction in the number of increments can translate to substantial savings in spectrometer time [8,87].

**Fig. 4:**
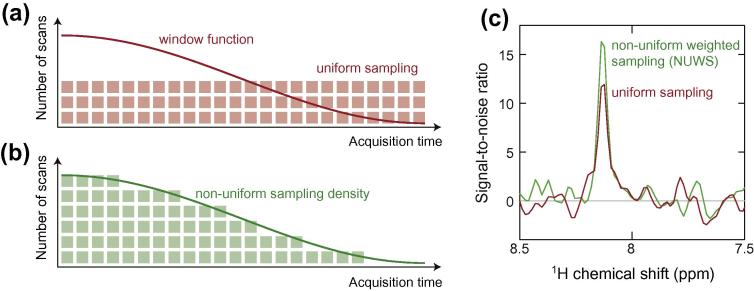
(a) Uniform and (b) non-uniform weighted sampling schemes for the acquisition of the indirect dimension of multidimensional spectra, illustrating the relationship between the window function, applied to uniformly sampled data, and the sampling density function applied during NUWS acquisition [24,25,92]. (c) Comparison of the sensitivity of uniform and NUWS sampling schemes. One-dimensional cross-sections through ^1^H,^15^N-SOFAST-HMQC spectra of α-synuclein (5 μM, 277 K, 700 MHz), recorded with identical experiment durations and acquired/processed with cosine-squared sampling densities/window functions respectively. A 23% increase in sensitivity was obtained with the NUWS scheme. Adapted from [26,27,92].

Multidimensional NMR data is most commonly acquired with uniform sampling of the Nyquist grid, followed by apodization and processing with the discrete Fourier transform. The selection of an appropriate window function is important and has a large effect on the ultimate SNR of the spectrum. An alternative approach is the use of non-uniform sampling (NUS) methods, whereby the Nyquist grid is undersampled and advanced computational techniques are employed to reconstruct the frequency domain spectrum [9–11,88,89]. In the sampling-limited case, where the SNR of individual points is high, NUS approaches can provide order-of-magnitude savings in spectrometer time for the acquisition of multidimensional spectra. In the case of dilute samples such as RNCs, where many scans must be accumulated for every point in the indirect dimension, NUS methods have been reported to increase experimental sensitivity [12,13,90]. However, given the non-linear methods employed in the processing of NUS data, it is not straightforward to define the SNR of NUS spectra and alternative, indirect measures have been suggested, such as the probability of detecting weak peaks [14,91]. An alternative to NUS, applicable in the sensitivity-limited case where many scans are acquired for each point in the indirect dimension, is to sample every point in the Nyquist grid with a variable number of scans, according to some weighting function (Fig. 4a and b). This approach, termed non-uniform weighted sampling (NUWS), is an intermediate between uniform sampling and non-linear NUS methods, and data may be processed and their sensitivity analyzed in a conventional manner via the discrete Fourier transform [15–18,92]. However, while some reports have suggested sensitivity increases of 100–200% may be obtained from this approach [5,6,19,93,94], more recently we have compared the sensitivity of uniformed sampled and NUWS spectra on a like-for-like basis, accounting for an intimate connection between the acquisition sampling scheme and the apodization window function, and we find that the ‘real world’ sensitivity increase is only ca. 10–20% (Fig. 4c) [20–22,92]. Nevertheless, the NUWS approach may be readily implemented and accurately preserves peak intensities and lineshapes [23,92], and so it may yet provide a useful contribution to the study of RNC samples by NMR.

#### 3.3.3 Optimization of transverse relaxation

Up to this point, the discussion of sensitivity has focused on the choice of acquisition time and the apodization and sampling strategies. However, losses due to transverse relaxation are also extremely significant, both during chemical shift encoding periods (Eq. (3)) and also during magnetization transfer delays. This is strongly affected by the choice of nucleus and labeling scheme, but also by the particular *R*_2_ relaxation rates of the various coherences selected by the pulse program being executed. The choice of isotopic labeling schemes has already been discussed above (Section 3.1), and so the discussion here shall focus primarily on the optimal choice of pulse programs.

A large variety of pulse programs are available for the observation of ^15^N-labeled samples. The majority of published RNC studies have employed the SOFAST HMQC experiment (a longitudinally-optimized variant of the HMQC, discussed further in the following section) [28,29,36,38,39,95]. In principle, the HMQC experiment might be expected to be less sensitive than a sensitivity-enhanced HSQC, due to the factor of √2 arising from sensitivity enhancement in the HSQC, and the effect of ^1^H–^1^H couplings active during the multiple-quantum evolution period in the HMQC experiment. However, an empirical comparison of their sensitivity, for the proteins ubiquitin (8.6 kDa, 298 K) and SiR-FP18 (18 kDa, 298 K), found that the SOFAST HMQC had a sensitivity approximately 50% greater when compared to longitudinally-optimized HSQC experiments, which was attributed to greater losses in HSQC experiments arising from pulse imperfections and *B*_1_-field inhomogeneities [30,95].

An alternative route to maximizing the sensitivity of ^15^N-based experiments is through experiments that take advantage of cross-correlated relaxation effects, both to minimize tranverse relaxation rates (the TROSY effect) and to increase the efficiency of magnetization transfer steps (CRINEPT). The TROSY effect, in a ^1^H–^15^N spin system, arises from destructive interference between cross-correlated dipolar and chemical shift anisotropy (CSA) relaxation mechanisms [31,32,96]. The sign of the interference effect (constructive/destructive) is dependent upon the ^1^H and ^15^N spin-state, but in fully decoupled experiments these spin states are rapidly exchanged, and so their associated relaxation rates are averaged. For small proteins, this loss in sensitivity due to faster transverse relaxation is more than compensated for by the gain in intensity that results from collapsing the multiplet structure. In contrast, for larger proteins it is preferable to avoid decoupling (in one or both dimensions) and instead record spin-state selective spectra, in which only the slowest relaxing coherence is observed. The use of such TROSY methods has permitted high-resolution studies of extremely large systems, such as the 800 kDa GroEL chaperone (*τ*_c_ approximately 310 ns at 308 K) [33,97]. Further sensitivity gains may be obtained through the use of CRINEPT polarization transfer elements, which exploit cross-correlation relaxation mechanisms to enhance the transfer of polarization from *H_x_* to *H_x_N_z_*. [34,35,98,99]. A number of 2D CRINEPT/TROSY experiments have been proposed, which vary in their combination of polarization transfer steps and spin-state selectivity, reflecting various trade-offs between the length and complexity of the pulse sequence, loss in signal due to spin-state selectivity, and loss in sensitivity and resolution due to non-optimal transverse relaxation [36–40,97,98]. The greatest sensitivity was found for the [^15^N,^1^H]-CRINEPT-HMQC-[^1^H]-TROSY experiment [34,97], and this experiment has been successfully used to observe fast-relaxing folded resonances in an SH3 RNC system [40,41] (discussed further in Section 4). This experiment utilizes CRINEPT transfers but is ^1^H-decoupled during the indirect evolution period and hence both slow and fast-relaxating spin states are preserved. In contrast, the direct dimension is acquired without decoupling resulting in a doublet structure, but for large molecules, only the slowly-relaxation TROSY coherence is observable. The lack of spin-state selectivity results in a high sensitivity for this experiment, but in more flexible regions of the RNC the anti-TROSY line may not be completely suppressed by its rapid relaxation, and this can result in increased overlap of peaks within crowded spectra [6,40,42].

In the case of ^13^C-based observations of side-chain methyl groups (particularly favorable for folded nascent chains, Section 3.1), HMQC sequences are the preferred class of experiment as the sequence is fully optimized for the methyl-TROSY effect. In analogy to dipolar/CSA interference in the ^15^N TROSY effect, the transverse relaxation rate of some methyl coherences is also greatly decreased by the field-independent cancellation of intra-methyl dipolar fields [43,70]. To maximize the sensitivity gain, pulse sequences must minimize the mixing of these slow and fast relaxating coherences. The HMQC experiment is fully optimal in this regard, and therefore for slowly tumbling systems the sensitivity may be substantially greater than HSQC-based experiments [44–47,100]. Moreover, in contrast to HSQC experiments, Ernst angle excitation may be employed, and a fully longitudinal relaxation optimized experiment, the methyl SOFAST HMQC [48,95,101], has been successfully employed for the observation of RNC side-chain methyl groups [37,38,49].

Finally, although not so far employed in the study of RNCs, fractional deuteration or perdeuteration of samples would be expected to greatly improve the sensitivity in all cases, by elimination of unwanted dipolar relaxation pathways. Therefore, despite the greater expense of sample preparation, we expect that this may be an important avenue to explore in future studies.

#### 3.3.4 Optimization of longitudinal relaxation

As discussed in the previous section, the sensitivity of macromolecular NMR is limited by the rapid tranverse relaxation of the observed signal. However, the slow recovery of longitudinal magnetization also has a significant effect on experimental sensitivity. Indeed, as longtitudinal relaxation is substantially slower than transverse relaxation, the bulk of spectrometer time is allocated to the inter-scan recovery delay, during which no signal is being actively acquired. Since the earliest days of pulsed NMR, the choice of this delay, *T*_rec_, and the excitation flip angle, α, has been recognized to affect the sensitivity of the NMR experiment (signal-to-noise per unit time) [50–53,102]. For a fixed excitation angle of 90°, the optimum relaxation delay is 1.26*T*_1_. Ernst angle excitation, where the scan time is reduced and the excitation flip angle is set to an optimum value, cos *α* = exp(−*T*_rec_/*T*_1_), can provide a small additional sensitivity gain in compatible pulse programs, such as the HMQC. This may be of the order of 10% and increases as the scan time is decreased, reflecting a net gain in SNR from the accumulation of many individually noisy scans. Ultimately, the repetition rate is limited either by the combined length of the pulse sequence and the acquisition time, or by restrictions on the spectrometer duty cycle.

A more recent trend in pulse sequence design has been the acceleration of longitudinal relaxation through the use of selective pulses, wherein only the observed subset of spins are excited [31,32,95,103]. This preserves a bath of cold spins, the effect of which is to accelerate the longitudinal relaxation of the observed spins through cross-relaxation pathways. The return to equilibrium is therefore described by an effective relaxation time, *T*_1eff_, which may be significantly shorter than the *T*_1_. For example, in the case of ubiquitin a reduction in *T*_1eff_ from 0.9–1.4 s to 0.3–0.4 s was observed upon selective excitation of amide protons [54,55,95]. In larger, fractionally deuterated or perdeuterated molecules however, many fewer spins are present to act as a heat sink, reducing the effectiveness of band-selective methods, but this may be at least partially counterbalanced by the increased rate of cross-relaxation within slowly tumbling molecules, again resulting in useful sensitivity gains [36,101].

In the past several years, a variety of longitudinally-optimized experiments utilizing band-selective excitation have been described, including the LHSQC [53,103], SOFAST-HMQC [56,95,104], methyl-SOFAST-HMQC [57–59,101], the BEST family of 3D experiments [31,105,106] and BEST TROSY experiments [60,107,108]. The improvement in sensitivity in these experiments is dependent upon the sample and experimental conditions, but gains of a factor of 1.5–2.5 are commonly obtained. In particular, the increased sensitivity of both ^15^N and ^13^C SOFAST-HMQC experiments has been instrumental in a number of RNC studies [36–39,61]. The more recently described BEST TROSY experiments [61,107] may offer advantages for future studies, however, due to the decreased transverse relaxation rate (as discussed above) but also to an additional strategy for the enhancement of longitudinal polarization [62,108]. TROSY experiments, due to their spin-state selectivity, are able to merge the initial polarization of both protons and nitrogens resulting in a small sensitivity gain of 10–20% [62,63,109]. Additionally, *H_z_* magnetization arising from longitudinal relaxation during the chemical shift encoding delay is converted to −*N_z_* by the ST2-PT transfer element [64,109]. This can be inverted to *N_z_* following the acquisition period, and in BEST TROSY experiments where the inter-scan delay is short this additional polarization may survive and contribute substantially to the observed signal [65,108]. However, as the recovered polarization arises from relaxation during the chemical shift encoding delay, the sensitivity gain will not be constant, but rather will be manifested indirectly as a reduced apparent *R*_2_ relaxation rate in the indirect dimension. As discussed earlier, and according to Eq. (2), this will result in increased SNR in the resulting spectrum.

Finally, an alternative approach to longitudinal relaxation enhancement is the doping of the sample with a paramagnetic ion or complex. Gd^3+^ complexes were originally used to accelerate ^13^C longitudinal relaxation in directly-detected experiments [54,55,110], and later to reduce the *T*_1_ of solvent water protons, resulting in sensitivity gains for labile amides [55,111]. However, proton transverse relaxation rates are also greatly increased in the presence of Gd^3+^, resulting in line broadening and decreased sensitivity. A solution to this problem was found in the use of paramagnetic species such as Ni^2+^ with very short electronic relaxation times, under which conditions the effect on *R*_1_ and *R*_2_ rates is identical. As *R*_1_ ≪ *R*_2_ in the macromolecular limit, such paramagnetic species may have a substantial effect on longitudinal relaxation while affecting resonance linewidths only marginally [54,112]. Moreover, this approach may be combined with band selective excitation and rapid recycling experiments such as the SOFAST-HMQC, as discussed above, and in this way sensitivity improvements of a factor of ca. 1.5–2 have been reported [36,113]. While this approach has not yet applied to the study of RNCs (and indeed the compatibility of paramagnetic agents with the ribosome would need to be carefully assessed), the potential increase in sensitivity is highly attractive.

### 3.4 Application and optimization of NMR diffusion measurements

A critical measurement in the study of RNCs by NMR is the continual assessment of the diffusion coefficient, *D*, of the species being observed. NMR diffusion measurements were first employed in high-resolution NMR studies of the ribosomal L7/L12 stalk complex, to test and confirm that the observed species did in fact arise from ribosome-bound molecules rather than from released or degraded protein (Fig. 2e) [54,57], and the use of such measurements has remained essential in studies of RNCs [36,38,54,55]. At the heart of this approach is the difference in hydrodynamic radius, *r*_h_, between small proteins in free solution, typically 2–3 nm, and the 2.3 MDa 70S ribosome, 12.6 nm [55,114]. Given the Stokes–Einstein relation, *r*_h_ = *k*_B_*T*/6*πηD*, where *k*_B_ is the Boltzmann constant, *T* is the temperature, and *η* is the solution viscosity, this is equivalent to an approximately 4-fold difference in the translational diffusion coefficient, and this may be readily distinguished using pulsed-field gradient stimulated-echo (PFG-STE) NMR diffusion measurements [54,115].

The use of NMR for the study of translational diffusion is a rich field, with diverse biomolecular applications from the study of protein conformation [60,116] and protein–ligand interactions [66–69,117,118] to high-resolution in-cell NMR spectroscopy [36,38–40,119], and a number of excellent books are available describing the subject [37,120,121]. Here, we will focus on three aspects of NMR PFG diffusion measurements central to the study of RNCs: the ability to discriminate released material from ribosome associated nascent chains; selectivity for nascent chain resonances; and the sensitivity of the experiments.

The signal intensity in a PFG-STE experiment, *I*(*G*), is described as a function of the gradient strength, *G*, by the Stejskal–Tanner equation [70,115]:

(4) $$I(G)=I(0)\exp(-\gamma^{2}G^{2}\delta^{2}\sigma^{2}\Delta D)$$

where *γ* is the gyromagnetic ratio of the nucleus on which position is encoded, *δ* is the length of the encoding and decoding gradient pulses, *σ* is the shape factor of the gradient pulses, and *Δ* is the length of the diffusion delay between encoding and decoding gradients. Corrections to Eq. (4) for finite gradient pulse lengths and bipolar gradients have been omitted, as these may vary with the pulse program being employed [71,72,115,122–124].

The lifetime of an RNC sample is limited both by the rate of nascent chain release from the ribosome, and by the degradation of the ribosome itself. In our experience, nascent chain release is often more rapid than ribosome degradation, and so while relatively strong resonances may be observed from the L7/L12 stalk complex, ribosome diffusion is not a sufficiently reliable probe to verify nascent chain attachment (although observation of ribosome breakdown is certainly indicative of nascent chain release). Therefore, to fully assess the sample integrity it is essential to measure the diffusion of the nascent chain specifically. This is greatly facilitated by the use of isotope-edited or heteronuclear diffusion experiments, which effectively eliminate unlabeled ribosomal resonances.

In the case of ^15^N-labeled RNC samples, the current experiment of choice is the ^15^N-XSTE experiment [36–38,122]. In this experiment, spatial encoding/decoding occurs on ^1^H spins during 5.4 ms INEPT transfer periods (1/2^1^*J*_NH_), which sandwich a ^15^N stimulated echo. Thus, the gradient encoding occurs on high-γ ^1^H spins and long gradient pulse lengths *δ* may be employed during the INEPT delays which, according to Eq. (4), results in high sensitivity to small displacements, essential for the accurate characterization of slow moving ribosomal species. Storage as *N_z_* in the stimulated echo clearly provides isotope editing, but also takes advantage of the longer ^15^N *T*_1_ to reduce longitudinal relaxation losses during the diffusion delay *Δ*, increasing the sensitivity of the experiment. An additional advantage, when compared with alternative ^15^N-edited ^1^H-stimulated echo experiments [73,74,125,126], is that the XSTE is not sensitive to amide hydrogen exchange during long diffusion delays. This effect is most significant for unfolded nascent chains, and may reduce the sensitivity or bias the measurement towards larger diffusion coefficients. Finally, although not so far employed in the study of RNCs, the use of longitudinal relaxation optimized experiments such as BEST-XSTE [76,77,123] or SORDID [31,124] may be of future interest in enhancing the sensitivity of XSTE experiments.

The diffusion of ^13^C-labeled RNC samples may also be measured using a ^13^C-XSTE experiment [78,122]. However, due to the larger ^1^*J*_CH_ coupling constant the INEPT transfer delay is significantly shorter than in the ^15^N experiment (3.5 ms compared to 5.4 ms), and this limits the maximum gradient pulse length, *δ*, to ca. 3 ms. Therefore, to maintain the ability to discriminate slowly diffusing species, large increases in the diffusion delay *Δ* may be required, with an associated loss in sensitivity due to increased longitudinal relaxation. As an alternative, a ^1^H-STE-^1^H, ^13^C-HMQC experiment may be acquired [79,127]. This experiment has a number of advantages over the ^13^C-XSTE. Firstly, there is no restriction on the gradient pulse length, *δ*, as spatial encoding/decoding does not occur during INEPT transfers, resulting in greater sensitivity to small diffusion coefficients. In contrast to amides, methyl protons are non-exchangeable and therefore no loss of sensitivity or accuracy arises from the use of ^1^H stimulated echoes. Finally, while losses to longitudinal relaxation are somewhat greater for the ^1^H-STE compared to the ^13^C-XSTE, transverse relaxation is significantly slower in the ^1^H-STE-HMQC experiment, as it is optimized for the methyl-TROSY effect with minimal mixing of slow and fast relaxing coherences [39,40,70,127]. Therefore, particularly for the broad resonances associated with folded RNC species for which ^13^C-based observation is most favorable, the ^1^H-STE-HMQC experiment may be expected to have significantly greater sensitivity than the ^13^C-XSTE.

## 4 Observations of co-translational folding using NMR spectroscopy

In this section, we present a systematic review of the five NMR investigations of RNCs in the literature at the time of writing. The first observation of a ribosome nascent-chain complex by NMR was reported in 2007 by Christodoulou and colleagues [36,38]. The gelation factor ddFLN (also known as ABP-120), from *Dictyostelium discoideum*, was selected as a model system [80,128]. This is a tandem repeat protein comprising an N-terminal actin-binding domain, followed by five highly conserved filamin domains having an immunoglobulin fold [38,40,129–131]. To model the progressive emergence and co-translational folding of the full-length protein from the ribosome, a fragment was designed comprising the two C-terminal domains, ddFLN5 and ddFLN6, minus the C-terminal beta-strand of ddFLN6. A cell-free transcription–translation system was employed using ^13^C,^15^N-labeled amino acids and a DNA construct lacking a stop codon to create a homogeneous population of stalled ribosomes, which were subsequently purified by sucrose gradient ultracentrifugation. NMR spectra were acquired using the ^15^N-SOFAST-HMQC experiment to maximize the experimental sensitivity [80,95], and the ^15^N-XSTE experiment [81,82,122] was used to measure the diffusion of the nascent chain and verify its attachment to the ribosome. Following acquisition, samples were incubated with 1 mM puromycin to induce release of stalled nascent chains, and the 62% increase in signal intensity that was subsequently observed provided further confirmation of the ribosome attachment. Finally, in addition to these spectroscopic measures, separate radiolabeled samples were prepared using [^35^S]-methionine and incubated alongside NMR samples to provide an orthogonal biochemical probe of sample integrity during the measurement timecourse.

The 2D HMQC spectra obtained for these RNC samples were analyzed by comparison with spectra of the released two domain construct, and the single domain ddFLN5, in the presence and absence of the ribosome particle. The RNC spectrum contained a number of poorly dispersed and highly overlapped resonances with proton chemical shifts between 8.2 and 8.6 ppm, characteristic of disordered states, but also a number of very low intensity dispersed resonances with proton chemical shifts between 6.5 and 10 ppm. These resonances overlapped closely with the spectrum of isolated ddFLN5, with only very small chemical shift changes of ca. 0.18 ± 0.17 ppm. This important result provided, for the first time, direct evidence that folded structures can be populated co-translationally, and that the folded structure is not significantly perturbed by the presence of the ribosome. The poorly dispersed resonances also overlaid well with spectra of the full-length construct in isolation, allowing them to be attributed to the ddFLN6 linker, which remained disordered both in isolation (due to the absence of the terminal beta-strand) and in the RNC, indicating that the presence of the ribosome did not strongly alter the folding equilibrium. An analysis of the ^1^H linewidths of cross-peaks determined average linewidths of 59 ± 26 Hz in the RNC, compared with 41 ± 11 Hz for the isolated construct in the presence of ribosomes, and some evidence was obtained for site-selective broadening in the N-terminal hemisphere of ddFLN5. Together, these observations indicate that the tumbling of the nascent ddFLN5 domain is dynamically decoupled from the tumbling of the ribosome through the flexibility of the disordered ddFLN6 linker region, but that there may still be weak and transient interactions between the nascent chain and the ribosome surface.

A second study of the same nascent chain system investigated the use of methyl groups to probe the structure and dynamics of side-chains within the nascent chain [37,83]. As discussed earlier, methyl groups in general have intrinsically higher sensitivity when compared to amides, and in this case the SNR was approximately 2.5 times greater for methyl resonances in a ^1^H,^13^C-SOFAST-HMQC relative to amide resonances in a ^1^H,^15^N-SOFAST-HMQC acquired under the same conditions. For very dilute systems such as RNCs, this is a highly significant gain in sensitivity. The observed RNC spectrum contained several well-resolved, dispersed resonances which overlaid closely with those of isolated ddFLN5, with extremely small proton chemical shift changes of 0.003 ± 0.014 ppm, again indicating that the nascent chain populates a folded state with a structure very similar to that observed for the free protein. As was the case in the ^1^H,^15^N spectrum, differential line broadening of methyl resonances was also observed, and following incubation with puromycin to induce release of the nascent chain, these peaks were found to increase in intensity. However, the most highly broadened methyl resonances were located in the core of the domain, in contrast to broadened amide resonances which were clustered in the N-terminal hemisphere of the domain. The reason for this discrepancy has not yet been fully rationalized, though further analysis with careful statistical consideration of measurements at very low SNR, will permit a more complete understanding of line broadening and dynamics within this RNC system.

The two studies discussed above used cell-free translation systems to produce the RNC samples. While this is a powerful and highly flexible approach allowing control over all aspects of isotopic labeling, the cost of regularly producing samples on the scale required for NMR is prohibitive. This stimulated the development of alternative *in vivo* approaches [38,39,84], using the 17 amino acid translational arrest motif derived from the *E. coli* secretion monitor protein, SecM (Section 3.2) [76,132,133], with subsequent purification by affinity chromatography and ultracentrifugation.

**Fig. 5:**
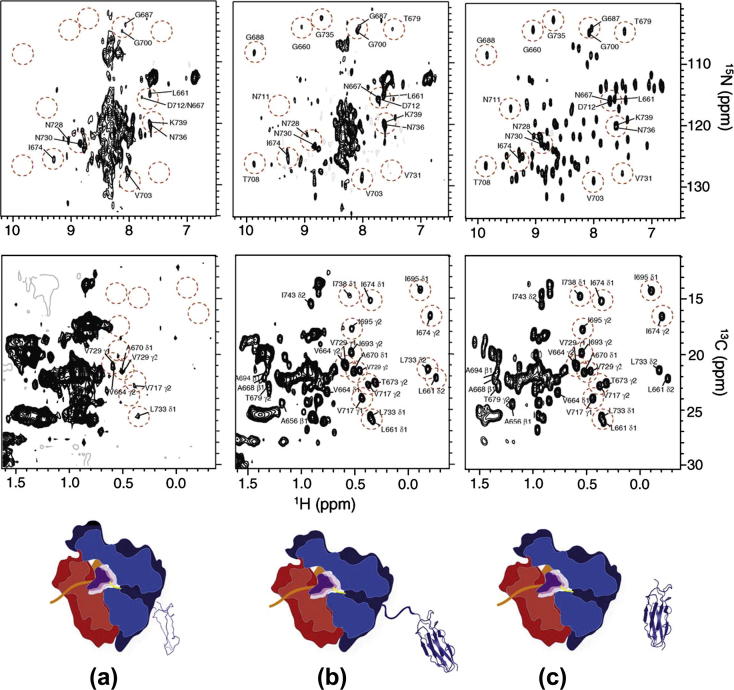
Observing the progressive folding of a ddFLN5 nascent chain by NMR. ^1^H–^15^N SOFAST-HMQC (top) and ^1^H–^13^C HMQC (bottom) spectra of (a) a folding-incompetent ddFLN5 RNC, in which the C-terminus of ddFLN5 is 21 aa from the PTC; (b) a folding-competent ddFLN5 + 6 RNC, in which an 89 residue fragment of ddFLN6 results in a total linker length of 110 aa; and (c) isolated ddFLN5. Spectra were acquired at 700 MHz, 298 K. Reprinted from [6], data taken from [38].

The first of the above studies [38] continued the characterization of the co-translational folding of the ddFLN protein. Two constructs were prepared comprising either ddFLN5 + 6 or the single ddFLN5 domain, fused to a C-terminal SecM motif and an N-terminal hexa-His tag. By passaging the cells selectively into a rich isotopically labeled medium for protein expression, ^13^C/^15^N-labeled nascent chains could be produced specifically against a background of isotopically silent ribosomes. A two-stage purification was employed selecting on the basis of molecular weight (sucrose gradient ultracentrifugation) and for the presence of the N-terminal hexa-His tag (metal affinity chromatography), to obtain a highly homogeneous population of stalled ribosomes carrying selectively labeled nascent chains. As previously, both ^1^H,^13^C and ^1^H,^15^N-SOFAST-HMQC spectra were acquired (Fig. 5). ^15^N-XSTE diffusion experiments were recorded to monitor the attachment of the nacent chain to the ribosome.

The comparison of the two RNC constructs provides a preliminary exploration of the dependence of co-translational protein folding on the length of the translated polypeptide, and in particular, the distance of the domain from the peptidyl-transferase center (PTC). In the shorter construct, the C-terminus of ddFLN5 is 21 residues from the PTC, while in the longer construct, the C-terminus of the domain is 110 residues from the PTC. By comparison, the ribosome exit tunnel encloses approximately 30 residues, and so in both constructs the terminal beta strand of ddFLN5 or ddFLN6 is not expected to be accessible for folding. In accordance with this, NMR spectra of the longer ddFLN5 + 110 RNC contained well-dispersed cross-peaks overlaying closely with the spectra of isolated ddFLN5 (Fig. 5b and c), in addition to a cluster of poorly resolved amide resonances with proton chemical shifts of 8–8.5 ppm, which were attributed to a disordered and flexible ddFLN6 linker. NMR spectra of the shorter ddFLN5 + 21 RNC were also typical of a disordered state (Fig. 5a), and cross-peaks in the ^1^H,^15^N HMQC spectrum overlaid well with a spectrum of isolated ddFLN5 denatured in 8 M urea.

In a parallel development and application of an *in vivo* sample preparation approach, the co-translational folding of the protein barnase was investigated by NMR [39]. The folding mechanism of barnase has been exceptionally well characterized in the isolated protein [134–136], and so NMR studies of co-translational folding in RNCs may be expected to yield a very interesting and informative comparison. Again, a SecM sequence was employed to create a stalled population of ribosomes, which were subsequently purified using a tobacco etch virus (TEV) protease-cleavable N-terminal Strep tag. A 50 residue sequence from the protein RpoB was incorporated between the barnase domain and SecM fragment to create a linker region, extending a total of 86 residues from the PTC, with sufficient flexibility to allow independent tumbling of the barnase domain and therefore observation by solution-state NMR. ^1^H,^15^N-SOFAST-HMQC spectra of the RNC were acquired, with excellent SNR, and 100 dispersed cross-peaks could be identified. Of these, 72 could be assigned by comparison with chemical shifts deposited in the Biological Magnetic Resonance Data Bank (BMRB) [137], and only small chemical shift changes were observed in the RNC (^1^H and ^15^N standard deviations of 0.04 ppm and 0.4 ppm respectively) indicating that the nascent chain is able to fold co-translationally to a highly native-like structure.

The final study to be discussed here is an investigation of the length-dependent folding of the SH3 domain from α-spectrin [40]. This is a small and moderately stable domain (63 residues, Δ*G*_F–U_ = 3.9 kcal mol^−1^ [138]) comprising five β-strands arranged in an orthogonal β-sandwich. The domain was previously studied as an RNC using biochemical methods, where protection of the nascent chain from proteinase K digestion indicated the formation of stable tertiary structure [139]. For the NMR study, an *in vivo* approach based on a 27 residue SecM-derived stalling motif was used to produce ^13^C/^15^N isotopically labeled RNC samples, using a cleavable N-terminal 3xStrepII-Smt3 tag as the basis for purification by affinity chromatography. A TEV protease cleavage site was also inserted at the C-terminus of the SH3 domain, upstream of a short linker sequence and the SecM motif, so in total the SH3 domain was 50 residues from the PTC. The integrity of the nascent chain was assessed by the progressive emergence of cross-peaks in the 2D spectra on timescales of >48 h, indicative of nascent chain release; and also by ^14^N and ^15^N-edited diffusion measurements, to characterize the hydrodynamic radii of both the nascent chain and the unlabeled ribosomes. Finally, an increase in the nascent chain diffusion coefficient was observed following TEV cleavage of the RNC, which provided an additional proof of ribosomal attachment. 2D NMR spectra were acquired using longitudinal relaxation optimized [^1^H,^15^N]-CRINEPT-HMQC-[^1^H]-TROSY (discussed in Section 3.3) and ^1^H,^13^C-HMQC experiments. The transverse relaxation optimized CRINEPT methodology is likely to have provided a useful increase in sensitivity, but there is also an increase in spectral overlap arising from the anti-TROSY line, particularly for more slowly-relaxing disordered resonances. Interestingly, a comparison of the relative intensities of the TROSY and anti-TROSY lines may in the future provide a simple, semi-quantitative probe of local rotational dynamics in the nascent chain complex.

2D NMR spectra of the SH3 RNC showed 22 amide and 23 side-chain resonances, distributed throughout the domain sequence, with chemical shifts very similar to those of the isolated domain (Δ*δ*_HN_ 0.040 ± 0.005 ppm, Δ*δ*_HC_ 0.007 ± 0.002 ppm); other resonances could not be distinguished due to overlap with ribosomal background signals. As was found for ddFLN5 and barnase, this indicates that the domain is able to fold co-translationally to a structure closely resembling that found in free solution. An analysis of amide proton linewidths determined average linewidths of 36 ± 2 Hz for the RNC, compared to 28 ± 1 Hz upon release and 23 ± 1 Hz for the free protein. This ca. 10 Hz increase in linewidth was compared to linewidths of over 1000 Hz that are expected for the 70S ribosome, indicating that less than 1% of the nascent chain is bound to the ribosome surface at a given moment (assuming fast exchange between ribosome-surface-bound and non-ribosome-surface-bound forms) and therefore that the ribosome is unlikely to strongly modulate the folding landscape of this protein through interactions with its folded state.

To compare the effect of ribosomal attachment on the (unobserved) unfolded state of the SH3 RNC, a protein engineering strategy was employed, introducing an ‘m10’ variant containing two destabilizing mutations that result in complete unfolding of the domain [140]. [^1^H,^15^N]-CRINEPT-HMQC spectra of the m10 RNC showed limited proton chemical shift dispersion, characteristic of a disordered state, and which overlaid closely with the free m10 protein, with negligible chemical shift changes. This indicates that the conformational sampling of the unfolded polypeptide is not significantly perturbed by the presence of the ribosome. Thus, the ribosome appears to be essentially inert to both the folded and unfolded states of the protein.

Finally, to investigate the length dependence of folding, in analogy to the study of ddFLN [38] two ‘folding snapshots’ were created in which 5 and 22 residues were truncated from the C-terminus of the domain. This resulted in effective linker lengths of 26 and 9 residues respectively from the PTC (compared to a linker length of 50 residues in the folded wild-type and unfolded m10 RNCs), and given that ca. 30 residues within an extended linker are expected to be buried by the ribosomal exit tunnel, these lengths correspond to the emergence of two or four of the five β-strands comprising the SH3 domain. 2D NMR spectra of the RNCs were acquired, and in both cases the spectra exhibited very limited chemical shift dispersion, indicating that the nascent chains remained disordered with no formation of stable tertiary structure. From this it was concluded that complete emergence of the SH3 domain is required before co-translational folding may occur. However, it should perhaps be noted that due to the 31 residue SecM motif employed in this study, the C-terminal residues were completely absent from the RNC construct, rather than being occluded by the exit tunnel, and that the truncated domains also failed to fold in free solution. Therefore, at this stage there is some difficulty in identifying what effect, if any, the presence of the ribosome has upon the folding of these truncations, but this will undoubtedly be an interesting question for future investigations to address.

## 5 Future prospects

### 5.1 Mapping the co-translational protein folding landscape

The NMR studies of RNCs discussed above have begun to address a very basic question: at what distance from the PTC can the formation of stable tertiary structure first occur? The answer to this question will depend on the structure adopted by the nascent chain within the ribosome exit tunnel [141,142]. The tunnel is 80–100 Å in length (depending on the definition of the exit point) and 10–20 Å wide [143], hence for a polypeptide in a fully extended conformation approximately 25 residues are expected to be occluded by the ribosome, while if a fully α-helical conformation were adopted as many as 50–60 residues might be occluded. We note however that while compact α-helical structure has been observed within the exit tunnel by cryoelectron microscopy, in general only short α-helical segments have been reported [31,77] and therefore in reality the figure of 50–60 residues is likely overestimated. In particular, the stalled SecM nascent chain has been observed to have an extended conformation, which is required in order to make a number of specific interactions with the inner surface of the exit tunnel [32].

**Fig. 6:**
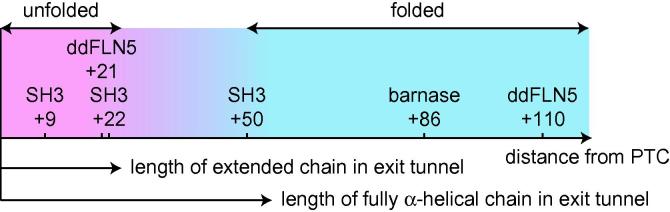
Summary of linker lengths for folded and unfolded RNCs observed by NMR spectroscopy [38,40,144], compared with the estimated number of residues enclosed by the ribosome exit tunnel for maximally extended and α-helical conformations [141–143].

Many experimental techniques have also been applied to characterize the length dependence of co-translational folding, such as the intrinsic fluorescence of the β-barrel protein eCFP, where a progressive increase in fluorescence was observed on increasing the linker length from 25 to 55 amino acids (measured from the PTC to the first structured residue of the β-barrel) [22]. Existing NMR studies have surveyed a wide range of linker lengths that separate folding competent domains from the PTC: 21 and 110 aa for ddFLN5 [38], 86 aa for barnase [39], and 9, 22 and 50 aa for SH3 [40]. While each protein system will clearly have its own peculiarities, it is nevertheless informative to compare the folding at the various lengths observed in these studies (Fig. 6). In no case is non-native structure observed (as probed by analysis of chemical shifts), and a folding transition is found to occur between 22 and 50 amino acids from the PTC, coincident with the emergence of the domains from the ribosome exit tunnel. At the resolution of current studies, however, it is not possible to determine the transition point more precisely, and therefore at present the NMR data are compatible with either extended or α-helical structure within the ribosome exit tunnel.

NMR is uniquely well-suited to characterizing the onset of folding, and to the broader enterprise of mapping the entire protein folding landscape as a function of chain length – the ‘co-translational protein folding landscape’ or folding funnel [41]. The measurement of chemical shift values gives a very direct probe of structure, allowing the identification of native structure, and the characterization of intermediate states. Moreover, as NMR is sensitive to folding kinetics through the effects of chemical exchange, such phenomena may in the future also allow the analysis of the kinetic barriers between folded, unfolded and intermediate states.

A complementary approach to the mapping of the co-translational folding landscape using stalled RNCs is through the analysis of isolated C-terminal truncations, mimicking the progressive emergence of domains from the ribosome exit tunnel. A number of systems have been studied in this way, using a variety of biophysical and spectroscopic methods including NMR together with circular dichroism (CD) spectroscopy, intrinsic fluorescence, and dye binding assay analyses [145–149]. Interestingly, a review of several proteins including CI2, barnase, staphylococcal nuclease and apomyoglobin observed that folding of fragments generally only occurred when almost the full chain length is present [150] (although the analysis of apomyoglobin fragments is complicated by their tendency to oligomerise [151]). For example, CI2, a small (64 amino acid) α + β domain that in isolation folds in a two-state manner, was found to populate a molten-globule-like state at intermediate lengths, and the final transition to its folded structure only occurred in the presence of the final two residues [146]. Changes in Hα chemical shift values were used to map the folding pathway on the structure according to the parameter $\Phi^{\text{NMR}}=(\delta_{\text{observed}}-\delta_{\text{random coil}})/(\delta_{\text{native}}-\delta_{\text{random coil}})$, where random coil chemical shift values were predicted from the local sequence [147]. This parameter, generally ranging between 0 and 1, is conceptually analogous to *Φ*-values used in the study of protein folding by protein engineering [152], and provides a simple measure of the structure of a residue in a given state relative to the native state. Its analysis in the case of CI2 determined that key stabilizing interactions present in the intermediate state were similar to those formed in the folding nucleus of the equilibrium folding pathway [147]. Given its simplicity, this parameter may in the future be a useful tool for the analysis of intermediate states observed within RNC samples.

The study of co-translational protein folding landscapes using series of isolated truncations provides an excellent starting point for systematic investigations of RNCs. Of particular interest will be the mechanisms by which the ribosome might perturb the landscape, as well as the magnitude of any effect (Section 5.2). Ultimately, we would like to understand folding as it occurs within the native environment of the cell [19], and the further perturbations to the folding landscape that might arise from macromolecular crowding effects and the presence of molecular chaperones (Section 5.3). The study of protein folding *in vivo* is being approached by many biophysical methods [21,153,154], but the emerging interest and development of methods for high-resolution NMR within living cells – ‘in-cell NMR’ – suggests that NMR spectroscopy also has the potential to make important contributions in this area [119,155–159].

### 5.2 Protein–ribosome interactions

Understanding the co-translational protein folding landscape, and in particular how it might be modulated by interactions with the ribosome itself, is crucial to understanding protein folding as it occurs within the cell. Preferential interactions with folded or unfolded states would provide a means by which the ribosome selectively stabilizes these states, in doing so affecting the position of the folding equilibrium, as well as the folding kinetics. Force microscopy studies of stalled ribosome bound nascent chain complexes have begun to investigate the effect of the ribosomal environment on the protein folding processes, and in the case of T4 lysozyme observed that the presence of the ribosome *decreased* the rate of folding [34]. This could be attributed to interactions of folded and unfolded states with the ribosome surface, at least part electrostatic in nature, acting to stabilize the states and so resulting in a larger free energy barrier between the states.

Solution-state NMR is exquisitely sensitive to interactions of proteins with the ribosome surface. Given the extremely slow tumbling of the ribosome, proton linewidths of the order of 1000–10,000 Hz could be expected for nascent chains interacting strongly with the ribosome, which is several orders of magnitude larger than observed for a small protein in isolation. This large difference in tranverse relaxation rates has been used to analyse the interaction of antibiotics with the ribosome [160]. In the context of RNCs, changes in resonance linewidths have been used to estimate the fraction of nascent chain interacting with the ribosome surface at any given instant, and in the case of the SH3 RNC discussed above, not more than 1% of folded or unfolded nascent chains were determined to be interacting with the ribosome [40]. Given the high sensitivity to low fractions of bound material, and as peak linewidths are readily measured even from noisy data, we expect that NMR will prove to be a useful tool for the further study of these interactions.

### 5.3 Interaction of nascent chains with trigger factor

**Fig. 7:**
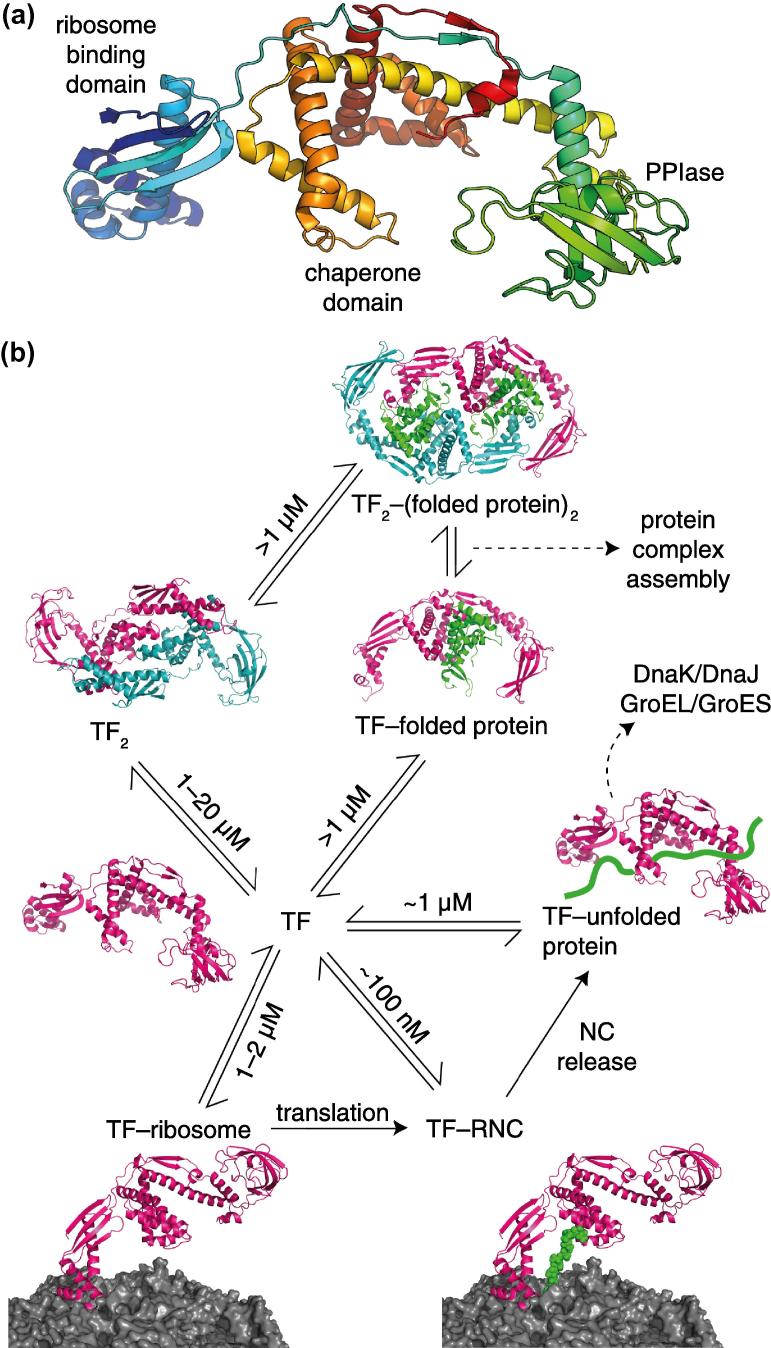
(a) Crystal structure and domain structure of *E. coli* trigger factor monomer [164] shown in ‘chainbow’ coloring (blue at N-terminal to red at C-terminal). (b) Schematic illustration of trigger factor binding equilibria (color code: TF chain(s), magenta and cyan; translated protein, green; ribosome, gray).

Trigger factor (TF) is a 48 kDa bacterial protein (Fig. 7a) that is the only molecular chaperone known to interact directly with the 70S ribosome [161–165]. It is ATP-independent, and present in the cytosol at a concentration of ca. 50 μM, in significant molar excess to the ribosome itself, which has an intracellular concentration of ca. 20 μM [166]. As such, it is most probably the first chaperone encountered by a nascent chain on emerging from the ribosome exit tunnel. Deletion of TF can be tolerated, although levels of the complementary chaperone DnaK are upregulated and the fraction of nascent chains interacting with DnaK increases from 15% to 40%. However, the combined deletion of TF and DnaK is lethal above 30 °C [167–169]. TF is involved in several binding equilibria (Fig. 7b): the free protein exists as a homodimer, with reported *K*_d_ values between 1 and 20 μM, but interacts with the ribosome in its monomeric form with a *K*_d_ of 1–2 μM and a residence time of ca. 10 s [170–172]. The presence of a nascent chain increases the ribosome binding affinity by an order of magnitude to ca. 100 nM, modulating both the association and dissociation rates of the complex [144,172]. TF has highly efficient peptidyl prolyl isomerase (PPIase) activity [173], but the primary function is thought to be as a ‘holdase’, interacting strongly with disordered hydrophobic and/or basic sequences to inhibit premature folding into misfolded states [27,144,172]. Any physiological function of the TF dimer itself is unclear at the present date, although both monomeric and dimeric TF have been found to interact with a number of folded proteins, including the ribosomal S7 protein, suggesting that TF may have a secondary role in assisting the assembly of protein complexes [174].

The TF monomer comprises three independently folding domains (Fig. 7a). The structure of the molecule has been determined crystallographically [164], but some NMR characterizations of domains have also been reported [175–177]. Overall, the structure forms a cradle over the ribosome exit tunnel, binding via the N-terminal domain to the L23 and L29 proteins and 23S rRNA of the large subunit [164,165]. This domain is also required for dimerization, and a 26 kDa homodimer of the NTD (TF_1–117_) has been studied by NMR and the backbone and side-chain chemical shifts fully assigned [178]. The central peptidyl prolyl isomerase (PPIase) domain (TF_151–251_) has an FKBP fold that was first determined by NMR, and an analysis of ps–ns backbone dynamics found that many residues were in exchange on a μs–ms timescale as had previously been observed for FKBP itself, indicating that dynamics were conserved in addition to structure [176]. The final domain is the C-terminal chaperone domain, which is implicated in substrate binding, although protein substrates may in fact bind to a variety of sites across the entire inner surface of the protein [164,179]. The structure of this domain has been solved crystallographically [164,180], but an NMR characterization was undertaken to resolve differences in subdomain orientation between these crystal structures [177]. However, observed residual dipolar couplings were not consistent with either structure. An analysis of ^15^N relaxation indicated the domain has substantial flexibility, suggesting that the conformation observed by RDCs may be an average of multiple conformations, arising from hinge motions of subdomains. Certainly, however, this provides a clear example where crystal structures do not represent the major conformation found in solution [177].

This brief summary has only scratched the surface of a fascinating chaperone system. Clearly, a series of careful and elegant biochemical and kinetic studies have yielded great insights into the chaperone mechanism of TF. However, a structural understanding of the interaction between the chaperone and its protein substrates is currently absent, and NMR could be a powerful source of information in this regard. From an NMR perspective TF is a complex system to study given the large molecular weight of TF and the ribosome, and the limited concentration of RNCs, but at a basic level the analysis of chemical shift changes and line broadenings in the presence of TF appears to be a reasonably feasible goal. The complete analysis of such data, however, will require the unraveling of a challenging system of coupled equilibria – dimerization, ribosome binding, and substrate binding – but the eventual results will surely be well worth the effort.

### 5.4 Interfacing with molecular dynamics simulations

The direct simulation of proteins using molecular dynamics (MD) methods offers, with the appropriate analysis, validation and visualization, deep insights into protein dynamics, folding and function [181]. While MD has historically been applicable to very simple systems over short timescales, the exponential growth in computational power means that the simulation of complex systems over biologically relevant timescales is becoming an increasingly realistic prospect [182]. At the present time, it is feasible to run all-atom simulations of small protein domains (∼10^4^ atoms) on timescales of up to a few ms [183–185], while simulations of the 70S ribosome (3 × 10^6^ atoms) have provided a glimpse of dynamics on timescales of 100 ns [186]. Indeed, the rate of improvement in biomolecular MD has outpaced Moore’s Law [182], due in part to the continual improvement in algorithm design, but also to the development of specialized hardware dedicated to the evaluation of molecular potentials [183,187]. At the current rate of progress, by 2030 it may become feasible to conduct all-atom simulations of the ribosome on the biologically important millisecond timescale [182].

In the interim, a variety of simplified, coarse-grained simulation methods have been developed in order to model large and complex systems such as the ribosome and the process of co-translational protein folding [188]. Elcock describes a Gō-type model [189] of the coupled protein synthesis and folding of CI2, barnase and Semliki forest virus protein (SFVP) [190]. The energetics of the model were calibrated using the experimental studies of C-terminal truncations of CI2 and barnase discussed above (Section 5.1), and the simulation results closely matched both the experimentally observed length dependence of folding, and the increased co-operativity of thermal unfolding observed near the full chain length. This validated simulation method was then used to explore the coupling of synthesis to folding. The ribosome was not observed to promote folding relative to the isolated truncations, and the folding pathways were not affected by the presence of the ribosome. In the case of the two-domain protein SFVP however, significant differences were observed in the *de novo* folding of the domain compared to the reversible folding in free solution: in particular, the emerging C-terminal domain was found to accrete onto the folded N-terminal domain. A particular virtue of MD methods is that simulation parameters may be varied to non-physical values to explore the properties of the system in more detail. By artificially increasing the stability of the CI2 domain in this manner, partially folded intermediate states were observed to become trapped within the ribosome exit tunnel, suggesting that delaying the folding of domains until the C-terminus has emerged may confer evolutionary advantages [190].

Further molecular dynamics studies, also using coarse-grained Gō-type models, have continued to investigate the length dependence of co-translational folding. Based solely on steric constraints, it was observed that native and non-native elements of tertiary structure can readily form within the ribosome vestibule (the final 20 Å of the 100 Å long ribosome exit tunnel) [191]. By analysis of the temperature dependence of RNC folding, perturbations to the free energy landscape due to the presence of the ribosome could be dissected into their enthalpic and entropic contributions, while analysis of RNC folding kinetics following a temperature quench enabled an analysis of folding pathways [192]. In contrast to earlier studies of CI2 and barnase [190] here it was observed, for a variety of small protein domains, that ribosomal attachment did affect the folding pathway, decreasing the diversity of the transition state ensemble and favoring the initiation of folding via N-terminal residues [192].

The differences between these simulation results highlight the continued importance of experimental observations in calibrating and validating the predictions of computer models. An extremely fruitful avenue in this regard is the combination of MD simulations with experimentally-based restraints. Chemical shift-based structure determination is a prime example of this approach, either through the use of chemical shift data to steer molecular fragment replacement methods [193–195], or as energy terms to bias forcefields in MD simulations towards the native state [196]. A particular advantage of these methods for RNC studies is that they appear to be relatively robust to sparse or missing data, as the available chemical shift data are combined with general protein behavior encoded via the simulation forcefield or fragment database [196,197]. Of course, the large number of atoms in the ribosome–nascent chain complex will impose significant computational demands. Nevertheless, given the complexity of RNC NMR, extracting the maximum information from the available spectra is an imperative, and in this regard the application of restrained MD appears a promising direction for future research.

### 5.5 Dynamical coupling of translation and folding

**Fig. 8:**
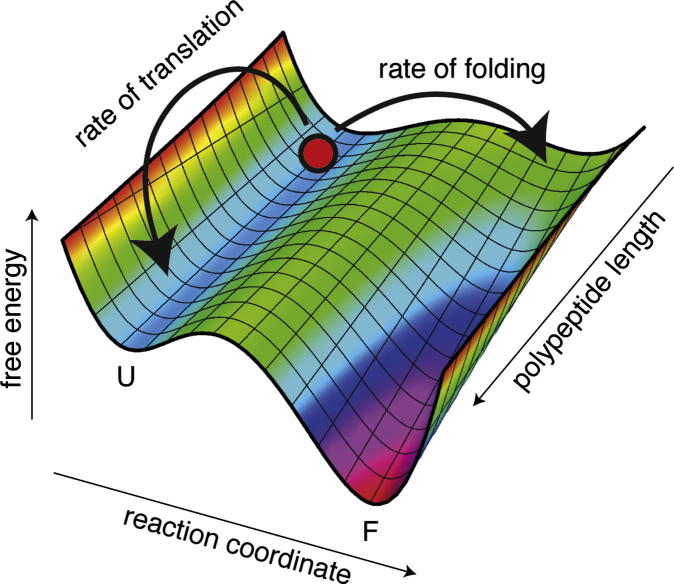
Schematic co-translational folding landscape, illustrating the changing stability of the states as a function of the polypeptide length, and the significance of the relative rates of translation and barrier crossing in determining the pathway of co-translational folding.

We close this review by considering once again the co-translational protein folding landscape. A fundamental difference between co-translational folding as it occurs within the cell, and as studied by NMR, is that co-translational folding in the cell is a kinetic process, potentially occurring far from equilibrium. By contrast, stalled RNC samples for study by NMR spectroscopy will have reached equilibrium during the lengthy expression and purification process. Thus, the states that may be kinetically accessed co-translationally may only be a subset of those that are observed in stalled nascent chains, and NMR observations may therefore chart the landscape, without necessarily determining the route that the nascent chain takes across it. This may be inferred, however, from a careful consideration of the kinetics of barrier crossing, relative to the rate of peptide synthesis (Fig. 8).

In the last few years, experimental evidence has begun to accumulate demonstrating that, at least in some cases, the coupling of translation to folding may be of biological significance [198,199]. Translation does not occur at a uniform rate, but is a stochastic process chained to the search for the cognate tRNA, and rare codons in the mRNA transcript may therefore introduce delays in translation and folding processes [200]. In particular, rare codons have been found to cluster in multi-domain proteins approximately 100 base pairs downstream of domain boundaries, a length corresponding to the complete emergence of the domain from the ribosome exit tunnel [201]. This suggests that these translation delays may be significant in allowing additional time for folding to occur, prior to synthesis of the following domain, reducing the probability of misfolding events. In support of this hypothesis, substitution of rare codons with common variants was found to decrease the folding yield [201], while up-regulation of low-abundance tRNAs promoted the aggregation of several cellular proteins [202]. ‘Silent’ single nucleotide polymorphisms to synonymous rare codons have also been implicated in altered gene function in mammalian cells [203].

If we compare the typical translation rate, *k*_trans_, to the rate of exchange between folded and unfolded states, *k*_ex_, we can categorize folding into three kinetic regimes. When folding is slow (*k*_ex_ ≪ *k*_trans_) the full length of the protein is expected to be synthesized and released from the ribosome before folding occurs, and hence the folding process will be identical to that that would be observed in reversible folding/unfolding studies of the isolated protein. By contrast, when folding is fast (*k*_ex_ ≫ *k*_trans_) the entire surface of the co-translational folding landscape may be sampled prior to release. In this regime, the co-translational acquisition of structure will be observed, but it may be of little consequence to the ultimate folding yield because the repeated unfolding and refolding will erase the memory of any co-translational effects. Therefore, it is the intermediate exchange regime in which biologically interesting behavior may emerge, and the effective co-translational ‘steering’ of the folding reaction might occur.

In bacterial cells the translation rate *k*_trans_ is approximately 15–20 amino acids s^−1^, while for eukaryotic cells it is approximately 1–5 amino acids s^−1^, and therefore the intermediate folding regime is likely to correspond to the slow to intermediate regimes of NMR chemical exchange, when *k*_ex_ is comparable to the difference in frequency between states, Δ*ω*. While systems in this regime are spectroscopically highly complex, particularly given the low sensitivity of RNC NMR, nevertheless it is under these conditions that translation can most effectively couple to the folding process to mimimise the sampling of states prone to misfolding. Deciphering the length-dependent folding of such RNC systems in thermodynamic, kinetic and structural detail by NMR will surely be a key challenge for the community in the years ahead.

## Glossary

BEST: band-selective excitation short-transient
CRINEPT: cross-correlated relaxation-enhanced polarization transfer
CTD: C-terminal domain
ddFLN: gelation factor (actin-binding protein 120) from *Dictyostelium discoideum*
EF-G: elongation factor G
EF-Tu: elongation factor Tu
FRET: Förster resonance energy transfer
HMQC: heteronuclear multiple quantum coherence
HSQC: heteronuclear single quantum coherence
IF2: initiation factor 2
INEPT: insensitive nuclei enhanced by polarization transfer
LHSQC: longitudinal relaxation optimized HSQC
MD: molecular dynamics
mRNA: messenger ribonucleic acid
NMR: nuclear magnetic resonance
NTD: N-terminal domain
NUS: non-uniform sampling
NUWS: non-uniform weighted sampling
PFG: pulsed field-gradient
PPIase: peptidyl prolyl isomerase
PTC: peptidyl transferase center
RDC: residual dipolar coupling
RNC: ribosome–nascent chain complex
rRNA: ribosomal ribonucleic acid
SecM: secretion monitor protein
SNR: signal to noise ratio
SOFAST: band-selective optimized-flip-angle short-transient
SORDID: signal optimization with recovery in diffusion delays
STE: stimulated echo
TEV: tobacco etch virus
TF: trigger factor
tmRNA: transfer-messenger ribonucleic acid
tRNA: transfer ribonucleic acid
TROSY: transverse relaxation optimized spectroscopy
XSTE: heteronuclear stimulated echo
